# Transcriptomic analysis on the effects of melatonin in gastrointestinal carcinomas

**DOI:** 10.1186/s12876-020-01383-z

**Published:** 2020-07-20

**Authors:** Lu Ao, Li Li, Huaqin Sun, Huxing Chen, Yawei Li, Haiyan Huang, Xianlong Wang, Zheng Guo, Ruixiang Zhou

**Affiliations:** 1 Department of Bioinformatics, Fujian Key Laboratory of Medical Bioinformatics, School of Basic Medical Sciences, Fujian Medical University, Fuzhou, 350122 China; 2 Department of Bioinformatics, Key Laboratory of Ministry of Education for Gastrointestinal Cancer, School of Basic Medical Sciences, Fujian Medical University, Fuzhou, 350122 China; 3 Department of Cell Biology and Genetics, School of Basic Medical Sciences, Fujian Medical University, Fuzhou, 350122 China; 4 Department of Human Anatomy, Histology and Embryology, School of Basic Medical Sciences, Fujian Medical University, Fuzhou, 350122 China

**Keywords:** Melatonin, Gastrointestinal carcinomas, Cell lines, Differentially expressed genes, Reverse, Functional enrichment analysis

## Abstract

**Background:**

Melatonin has been shown with anticancer property and therapeutic potential for tumors. However, there lacks a systematic study on the molecular pathways of melatonin and its antitumor effects in gastrointestinal carcinomas.

**Methods:**

Using the gene expression profiles of four cancer cell lines from three types of gastrointestinal carcinomas before and after melatonin treatment, including gastric carcinoma (GC), colorectal carcinoma (CRC) and hepatocellular carcinoma (HCC), differentially expressed genes (DEGs) and biological pathways influenced by melatonin were identified. The qRT-PCR analyses were performed to validate the effects of melatonin on 5-FU resistance-related genes in CRC.

**Results:**

There were 17 pathways commonly altered by melatonin in the three cancer types, including FoxO signaling pathways enriched by the upregulated DEGs and cell cycle signaling pathways enriched by the downregulated DEGs, confirmed the dual role of melatonin to tumor growth, pro-apoptosis and anti-proliferation. DEGs upregulated in the three types of cancer tissues but reversely downregulated by melatonin were commonly enriched in RNA transport, spliceosome and cell cycle signaling pathways, which indicate that melatonin might exert antitumor effects through these pathways. Our results further showed that melatonin can downregulate the expression levels of 5-FU resistance-related genes, such as thymidylate synthase in GC and *ATR*, *CHEK1*, *BAX* and *MYC* in CRC. The qRT-PCR results demonstrated that melatonin enhanced the sensitivity of CRC 5-FU resistant cells by decreasing the expression of *ATR*.

**Conclusions:**

Melatonin exerts the effects of pro-apoptosis and anti-proliferation on gastrointestinal carcinomas, and might increase the sensitivity of 5-FU in GC and CRC patients.

## Background

Melatonin (*N*-acetyl-5-methoxytryptamine), a hormone secreted by the pineal gland and gastrointestinal tract during night and daytime, plays a key role in circadian rhythms [1], antioxidant activities [2, 3] as well as immune system regulation [4, 5]. It has been reported that the melatonin concentration in the gastrointestinal tract tissues is 100–400 fold higher than that in plasma and liver is the main site for melatonin metabolism [6, 7]. Cumulative studies have suggested that some substances, such as dietary glycine, broccoli sprout, are associated with a reduced incidence of cancer [8, 9]. Decreased melatonin levels have also been demonstrated to be correlated with increased cancer risk. A large number of studies have reported that melatonin has anticancer effects on numerous types of tumors, such as liver [10, 11], colon [12], breast [13] and ovarian [14] cancers. These studies mainly highlight its dual role in tumor cells: pro-apoptosis and anti-proliferation, which are the two goals in the control of tumor growth. However, these in vitro studies only used tumor cell lines for a particular cancer type, and there lacks a systematic study to elucidate the global responsive pathways and the antitumor effects of melatonin’s actions across multiple tumor types.

Recently, there is an increasing interest in exploring the clinical application of melatonin in cancer therapy. Many studies suggested that melatonin treatment is useful in enhancing the efficacy of some chemotherapeutic drugs and controlling the progression of cancers [15–18]. For example, Lin et al. [19] found that melatonin synergistically promoted the sorafenib-induced apoptosis in hepatocellular carcinoma cell lines. Moreover, many studies demonstrated that melatonin is beneficial to reduce the side effects of chemotherapeutic drugs [20–23]. Lissoni et al. [22] found that melatonin attenuates the negative consequences of cisplatin in advanced non-small cell lung cancer patients. Therefore, it is worth to investigate the molecular mechanism of melatonin administration in aiding against different types of tumors.

The large-scale gene expression profiles facilitate us to characterize the association between melatonin and cancer development, therapeutic response. Gastric carcinoma (GC), colorectal carcinoma (CRC) and hepatocellular carcinoma (HCC) are three common malignant tumors in the digestive system, all with high morbidity and mortality across the world. In this study, our aim was to characterize the common biological signaling pathways altered by melatonin on the three types of gastrointestinal carcinomas with genome-wide expression data and further investigate the relationship between these pathways and the antitumor effect and synergistic drug response of melatonin.

We measured gene expression profiles of four tumor cell lines for the three cancer types treated with melatonin and analyzed differentially expressed genes (DEGs) between the treatment and control groups. Functional enrichment analyses showed that the DEGs after melatonin treatment in the three cancers were enriched in 17 common pathways, such as FoxO and ErbB signaling pathways enriched by the upregulated DEGs, and cell cycle signaling pathways enriched by the downregulated DEGs, confirmed its dual role in controlling tumor growth. We further found that the DEGs upregulated in tumor tissues but downregulated by melatonin in the cell lines were all enriched in RNA transport, spliceosome and cell cycle signaling pathways, which might be the potential targets for cancer therapy. We further compared the DEGs with 5-fluorouracil (5-FU) resistance-related genes in GC and CRC and found that melatonin might downregulate the expression levels of 5-FU resistance-related genes, such as thymidylate synthase (*TS*) in GC patients and *ATR*, *CHEK1*, *BAX* and *MYC* in CRC patients. The qRT-PCR results demonstrated the effect of melatonin by decreasing expression of *ATR* to increase the sensitivity of CRC 5-FU resistant cells. Our study is helpful to gain a comprehensive understanding of the effects of melatonin on gastrointestinal carcinomas.

## Methods

### Cell culture and reagents

The gastric adenocarcinoma cell line HGC-27, colorectal adenocarcinoma cell line HCT-8 and CRC 5-FU resistant cell line HCT-8/5-FU were grown in Roswell Park Memorial Institute (RPMI) 1640 medium (Hyclone, Logan, UT, USA.). The human hepatocellular carcinoma cell lines HepG2 and Huh-7 were cultured in Dulbecco’s modified Eagle’s medium (DMEM) (Hyclone, Logan, UT, USA.). All the cells were supplemented with 10% fetal bovine serum and maintained at 37 °C in 5% CO_2_. Cells were seeded in 9.6 cm^2^ culture dishes at a density of 1 × 10^6^ cells/well.

### Cell viability assays

GC cell line HGC-27 and CRC cell line HCT-8 were seeded into 96-well plates containing 100 μl medium at a density of 1000 cells/well. After 24 h incubation, cells were changed with fresh medium containing 0 (1% ethanol as control was added), 1, 2, 3, 4 or 5 mmol/L melatonin for 24 h, 48 h or 72 h. After the treatment, medium was discarded carefully and solution containing 20 μl MTS (CellTiter 96® AQueous One Solution Cell Proliferation Assay; Promega, Madison, WI, USA) and 80 μl serum free medium was added to each well and incubated for 2 h. Then the optical densities was measured at 490 nm with a microplate reader (Synergy HT; BioTek Instruments Inc., Winooski, VT, USA).

### RNA extraction and microarray expression analysis

The four tumor cell lines treated with 2.5 mmol/L melatonin for 24 h served as the treatment group and the rest cells cultured with ethanol served as the controls at the same time. RNA from the treatment group and the control group was extracted using the RNeasy Mini kit (Qiagen, Germany). The quality of RNA was measured using an Agilent 2100 Bioanalyzer (Agilent, USA). The fragmented cRNA for DNA microarray analysis was prepared according to the manufacturer’s instructions, then hybridized to customized Affymetrix GeneChip® PrimeView™ Human Gene Expression Array, which includes 49,495 probe sets representing 19,042 genes. Arrays were scanned with Affymetrix Genechip™ Scanner 30007G. Each sample had three biological replicates. Expression profiling data measured in our study are available in the Gene Expression Omnibus repository (GEO accession number: GSE132119).

### Quantitative RT-PCR analysis

For analysis of messenger RNA (mRNA) expression, reverse-transcription of cDNA was conducted using the ExScript RT-PCR Kit (Takara, Tokyo). Quantitative real-time (qRT-PCR) assays was performed using a SYBR Premix Ex Taq Kit (Takara, Tokyo) and the ABI StepOne Real-Time PCR System (Applied Biosystems). Cycle conditions were as follows: polymerase activation at 95 °C for 1 min, 40 cycles of denaturing at 95 °C for 15 s, and annealing/extension at 60 °C for 30 s. The relative expression of *ATR* was normalized to the expression level of glyceraldehyde-3-phosphate dehydrogenase (*GAPDH*), calculated by the 2-ΔΔCT method. The primer sequences are listed in Supplementary Table S1.

### Data pre-processing of expression data

Gene expression profiles of GC, CRC and HCC tumors and the corresponding normal samples used in this study were downloaded from GEO. The details of each dataset were shown in Table 1. The Robust Multi-array Average algorithm [30] were used to normalize the raw expression data. Probe-set IDs were mapped to Entrez gene IDs with their corresponding platform files. The expression value of a gene which was mapped to multiple probes was defined as the arithmetic mean of the expression values of those probes. Data were log2 transformed. Subsequent analysis was performed in R version 3.1.1.

**Table 1:** Datasets of cancer and normal samples for three types of gastrointestinal carcinomas

	GEO Accession	Platform	Normal Samples	Cancer Samples	References
GC	GSE27342	GPL5175	80	80	[24],
	GSE63089	GPL5175	45	45	[25],
CRC	GSE8671	GPL570	32	32	[26],
	GSE23878	GPL570	24	35	[27],
HCC	GSE14520	GPL3921	220	225	[28],
	GSE39791	GPL10558	72	72	[29],

### Identification of DEGs

The Student’s *t*-test was used to select DEGs between the treated and control cancer cell lines or between the cancerous and normal tissue samples. Because Student’s *t*-test biases towards genes with low expression levels in small size samples, i.e. the cancer cell line datasets here, the reproducibility-based pairwise difference (PD) [31, 32] was combined to detect DEGs between the treatment group and the control group of the cell line datasets. It has been demonstrated that the PD algorithm could identify many DEGs with high expressions in small-scale cancer cell line datasets which tended to be missed by Student’s *t*-test. The two DEGs lists detected by two algorithms were merged by excluding those with different dysregulated directions.

### Statistical analysis

A directed regulatory network of protein-protein interaction by linking DEGs of CRC cancer cell line HCT-8 with 85 genes related with 5-FU resistance in CRC [33, 34] was constructed in the SIGnaling Network Open Resource (SIGNOR) [35] database. The expression levels of 5-FU resistance-related genes are positively associated with the degree of drug resistance.

For analysis of IC_50_ value and the expression of *ATR*, significant differences were analyzed by independent sample *t*-test using SPSS software. Differences between groups were considered to be statistically significant at *p* < 0.05.

Functional enrichment analysis was performed based on the Kyoto Encyclopedia of Genes and Genomes [36]. The hypergeometric distribution model was used to determine biological pathways that were significantly enriched with DEGs [37]. The Benjamini and Hochberg procedure (BH) was used to adjust the *p*-values to control the False Discovery Rate (FDR) and the statistical significance was set as FDR < 10%.

## Results

### Melatonin inhibited cell growth of HGC-27 and HCT-8 cells

**Fig. 1:**
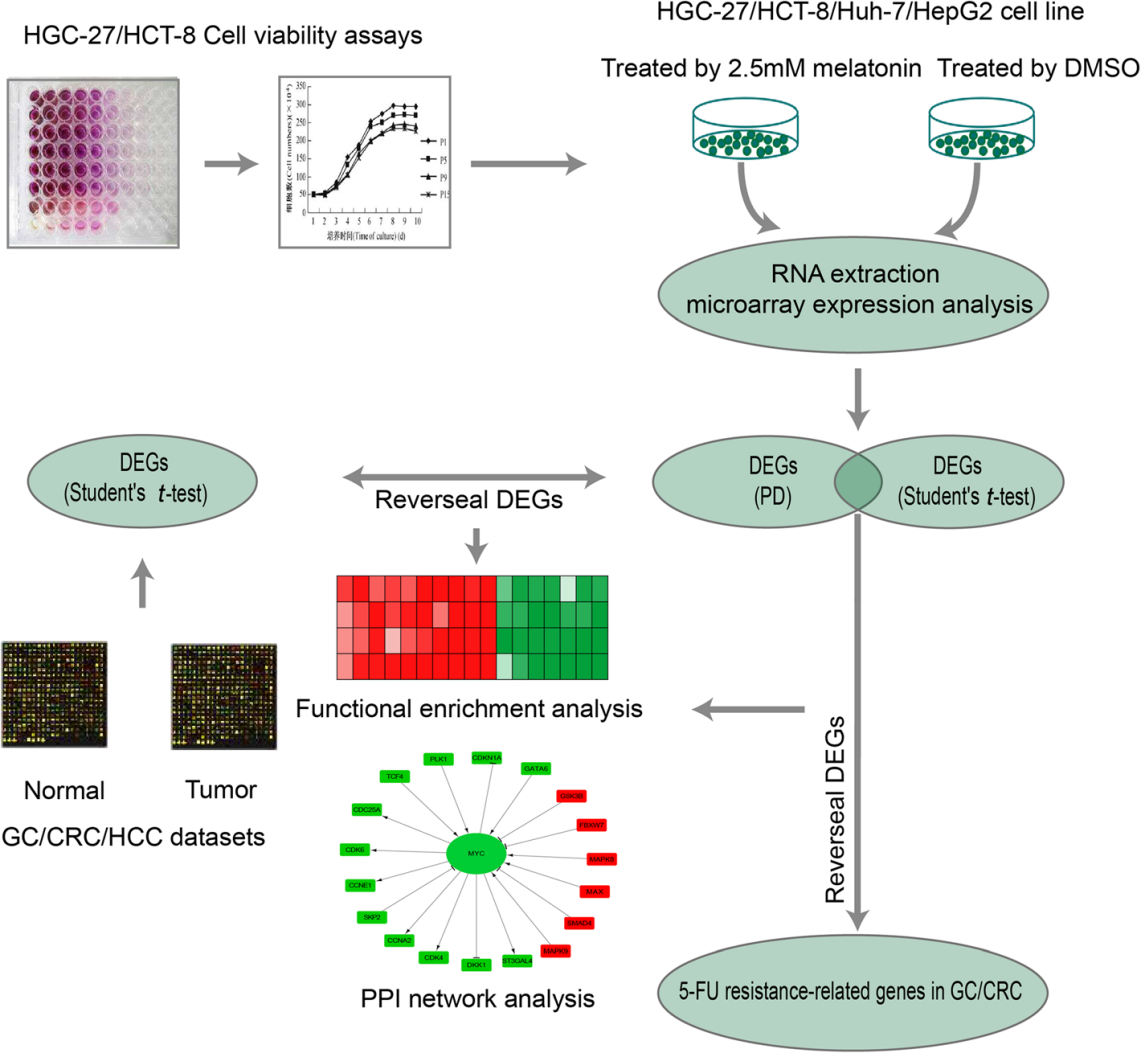
The flowchart of this study. First, Melatonin inhibited cell growth of HGC-27 and HCT-8 cells in a dose and time-dependent manner. The concentration (2.5 mmol/L) and time (24 h) of melatonin for treatment were determined. Second, four cancer cell lines (HGC-27, HCT-8, Huh-7 and HepG2) across three types of gastrointestinal carcinomas (GC, CRC and HCC) were treated by melatonin for 24 h and performed by DNA microarray analysis. Third, the DEGs by melatonin treatment detected by Student’s *t*-test and the reproducibility-based PD were combined to investigate the common biological signaling pathways altered by melatonin. Fourth, the DEGs detected between tumor and normal tissues but reversed by melatonin in cancer cell lines were used to explore the potential anticancer effects of melatonin. Finally, the 5-FU resistance-related genes in GC and CRC but reversed by melatonin in cancer cell lines were used to explore the potential of melatonin to increase the sensitivity of 5-FU in GC and CRC

**Fig. 2:**
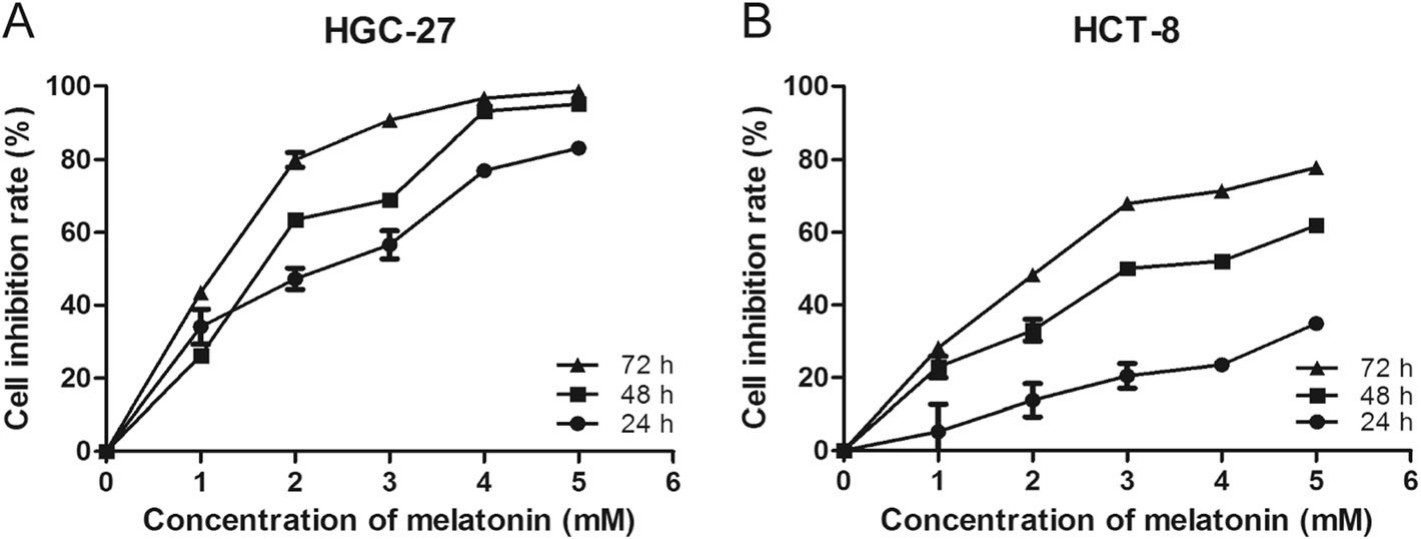
Effect of melatonin on cell growth in HGC-27 and HCT-8 cells. The antitumor effect of melatonin on GC cell line HGC-27 (**a**) and CRC cell line HCT-8 (**b**). The cells were treated with melatonin (0, 1, 2, 3, 4, 5 mmol/L) for 24 h, 48 h and 72 h, respectively. Cell viability was assessed by MTS assay

The flowchart was described in Fig. 1. GC cell line HGC-27 and CRC cell line HCT-8 were treated with 0, 1, 2, 3, 4 or 5 mmol/L melatonin for 24 h, 48 h or 72 h, respectively. Cell viability was assessed by MTS assay. The results revealed that melatonin inhibited the growth of HGC-27 and HCT-8 in a dose and time-dependent manner (Fig. 2). The melatonin concentrations of 50% inhibition of cell viability were 1.98 mmol/L and 8.82 mmol/L, respectively, for HGC-27 and HCT-8 for the 24 h treatment. At present, the consensus of melatonin concentration and exposure time for inhibiting the cell viability in HepG2 and Huh-7 cell lines is 1 mmol/L and 24 h, respectively [11, 15, 38, 39]. Based on these results, we selected 2.5 mmol/L and 24 h to treat the four tumor cell lines in the following experiments.

### Identification and functional analysis of dysregulated genes in cancer cell lines treated by melatonin

To provide a comprehensive overview of the common biological signaling pathways altered by melatonin on three cancer types, we first detected the DEGs in the cancer cell lines due to the melatonin treatment. Using Student’s *t*-test with 5% FDR control, 6236 DEGs were detected in the HGC-27 cell lines; if the reproducibility-based PD with a consistency threshold of 90% was used, 7287 DEGs were detected. A total of 5265 DEGs were commonly detected by the two methods, all of which were with the same dysregulated directions. Then the two DEGs lists were combined and a full list of 7898 DEGs of HGC-27 were obtained. Similarly, 6363, 10,282 and 7815 DEGs were detected in the HCT-8, Huh-7 and HepG2 cells, respectively (detailed information shown in Supplementary Table S2).

**Fig. 3:**
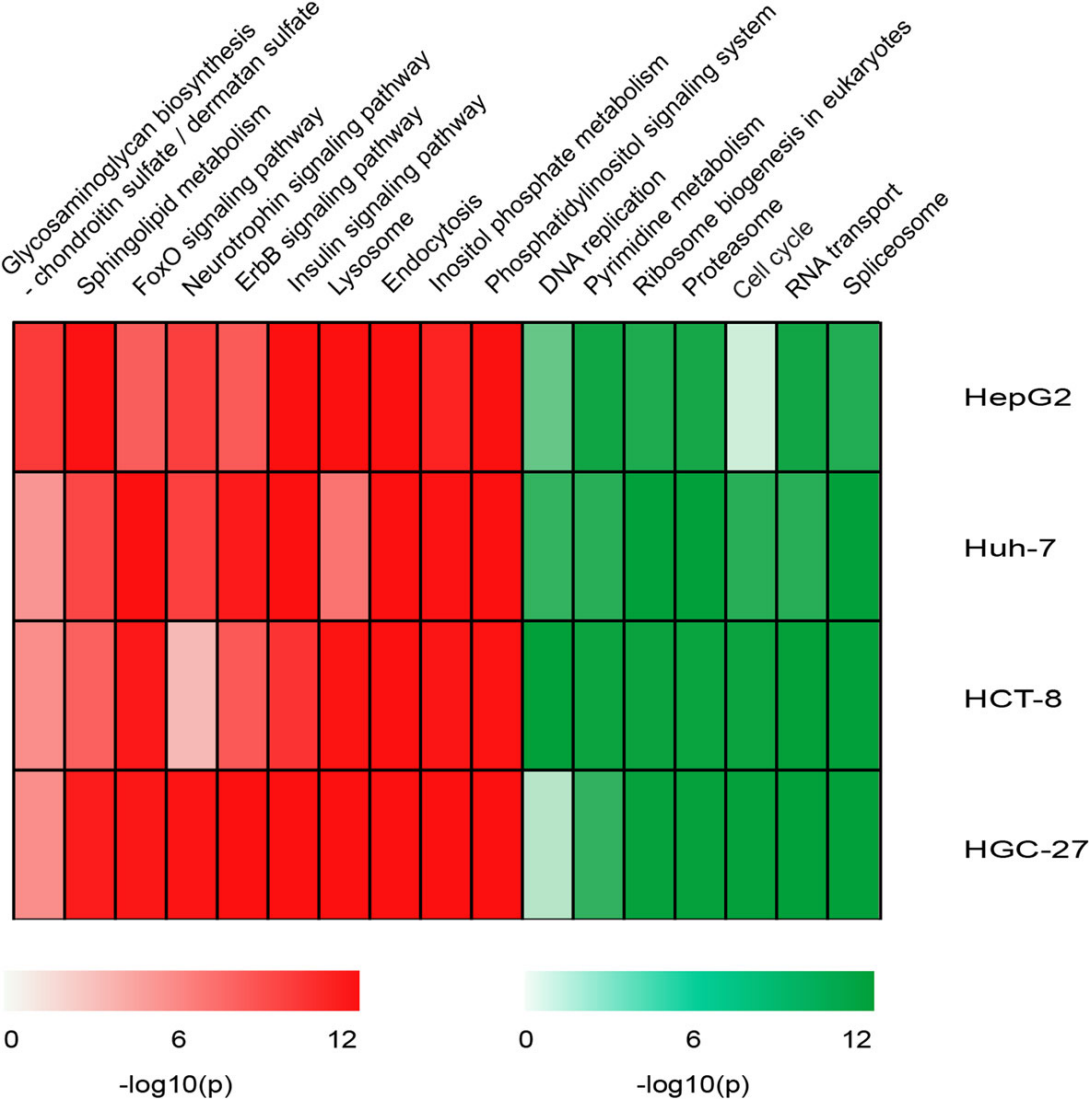
The common KEGG pathways significantly enriched by the upregulated and downregulated DEGs in four cancer cell lines treated by melatonin. The common KEGG pathways significantly enriched by the upregulated (Red) and downregulated (Green) DEGs in four cancer cell lines treated by melatonin. All *p* values of the KEGG pathway were adjusted by Benjamini and Hochberg (*p* < 0.1). -log10(*p*) was used to generate the heatmap

With a 10% FDR control, 4114, 3242, 4673 and 3837 upregulated DEGs of the four cell lines were enriched in 23, 44, 29 and 42 biological pathways, respectively (shown in Supplementary Table S3). There were 10 common pathways, including FoxO, ErbB and lysosome signaling pathways (Fig. 3). The FoxO family genes play a crucial role in tumor suppression by upregulating their target genes involved in apoptosis [40]. Our results also suggest that melatonin might enhance the apoptosis of tumor cells through the activation of FoxO signaling pathway [16].

Similarly, 3784, 3121, 5609 and 3978 downregulated DEGs of the four cell lines were enriched in 10, 14, 11 and 12 biological pathways, respectively (shown in Supplementary Table S4). There were 7 common pathways, including pyrimidine metabolism, DNA replication and cell cycle signaling pathways (Fig. 3). These results further support the view that melatonin reduces the cell cycle of tumor to control tumor growth [10, 15, 41].

### Comparison between the dysregulated genes in cancer tissues and those reversed by melatonin

To explore the potential anticancer effects of melatonin, we compared the DEGs found in cancer cell lines with those in cancer tissues. Using Student’s *t*-test with 1% FDR control, 3278 and 7459 DEGs were identified between GC cancerous and normal samples in GSE27342 and GSE63089, respectively. A total of 3068 DEGs with the same dysregulation directions in the two datasets were selected as dysregulated genes in the state of GC. Among the 1475 upregulated genes, 603 DEGs were downregulated in the HGC-27 cell lines treated by melatonin, and enriched in 5 biological pathways with 10% FDR control. Among the 1593 downregulated genes, 334 DEGs were upregulated by melatonin, which were enriched in 9 biological pathways (Supplementary Table S5 and S6).

Similarly, 3336 DEGs with the same dysregulation directions in dataset GSE8671 and dataset GSE23878 were identified in CRC tumors using Student’s *t*-test with 1% FDR control. Among the 1317 upregulated and 2019 downregulated genes in CRC tumors, 605 and 425 DEGs were reversely downregulated and upregulated in the HCT-8 cell lines treated by melatonin, respectively, which were enriched in 7 and 30 biological pathways. Moreover, 4257 DEGs with the same dysregulation directions in dataset GSE14520 and dataset GSE39791 were identified in HCC tumors using Student’s *t*-test with 1% FDR control. Among the 2865 upregulated genes, 1136 and 868 DEGs were downregulated, respectively, in the Huh-7 and HepG2 cell lines treated by melatonin, while among the 1392 downregulated gens, 355 and 271 DEGs were upregulated, respectively. The functional enrichment analysis results were shown in Supplementary Tables S5 and S6.

Interestingly, there were 4 common pathways enriched by those DEGs which were upregulated in three cancers but downregulated in all four cell lines treated by melatonin, including ribosome biogenesis in eukaryotes, RNA transport, spliceosome and cell cycle signaling pathways. These results suggest that melatonin might exert antitumor effects through these pathways.

### Comparison with the genes related with 5-FU resistance in GC and CRC

Because 5-fluorouracil (5-FU) is a routine chemotherapeutic agent of DNA damage in GC and CRC, we further investigated whether DEGs altered by melatonin are associated with 5-FU resistance.

Recently, we have developed a signature consisting of two gene pairs which could robustly predict the prognosis of GC patients treated with 5-FU-based chemotherapy [42]. Using Student’s *t*-test with 5% FDR control, 1969 DEGs were identified between 88 patients with high-risk and 35 patients with low-risk of resistance to 5-FU-based regimens. Among the 871 downregulated genes in the resistant high-risk GC patients compared with the low-risk patients, 234 DEGs were upregulated in the HGC-27 cell lines treated by melatonin. Meanwhile, among the 1098 upregulated genes in the resistance high-risk GC patients, 520 DEGs were downregulated in the HGC-27 cell lines treated by melatonin, which were enriched in 12 biological pathways with 10% FDR control (Supplementary Table S7). The pyrimidine metabolism pathway, which is responsible for the metabolism of 5-FU, was included, and the thymidylate synthase (*TS*) gene involved in the pathway was downregulated by melatonin. It has been reported that 5-FU exerts its anticancer effects through inhibition of *TS* to disrupt DNA synthesis and repair, resulting in lethal DNA damage [43]. Zembutsu et al. have revealed that there is an inverse relationship between mRNA levels of *TS* and 5-FU sensitivity in a panel of cancer cell lines, including GC cell lines [44].

**Fig. 4:**
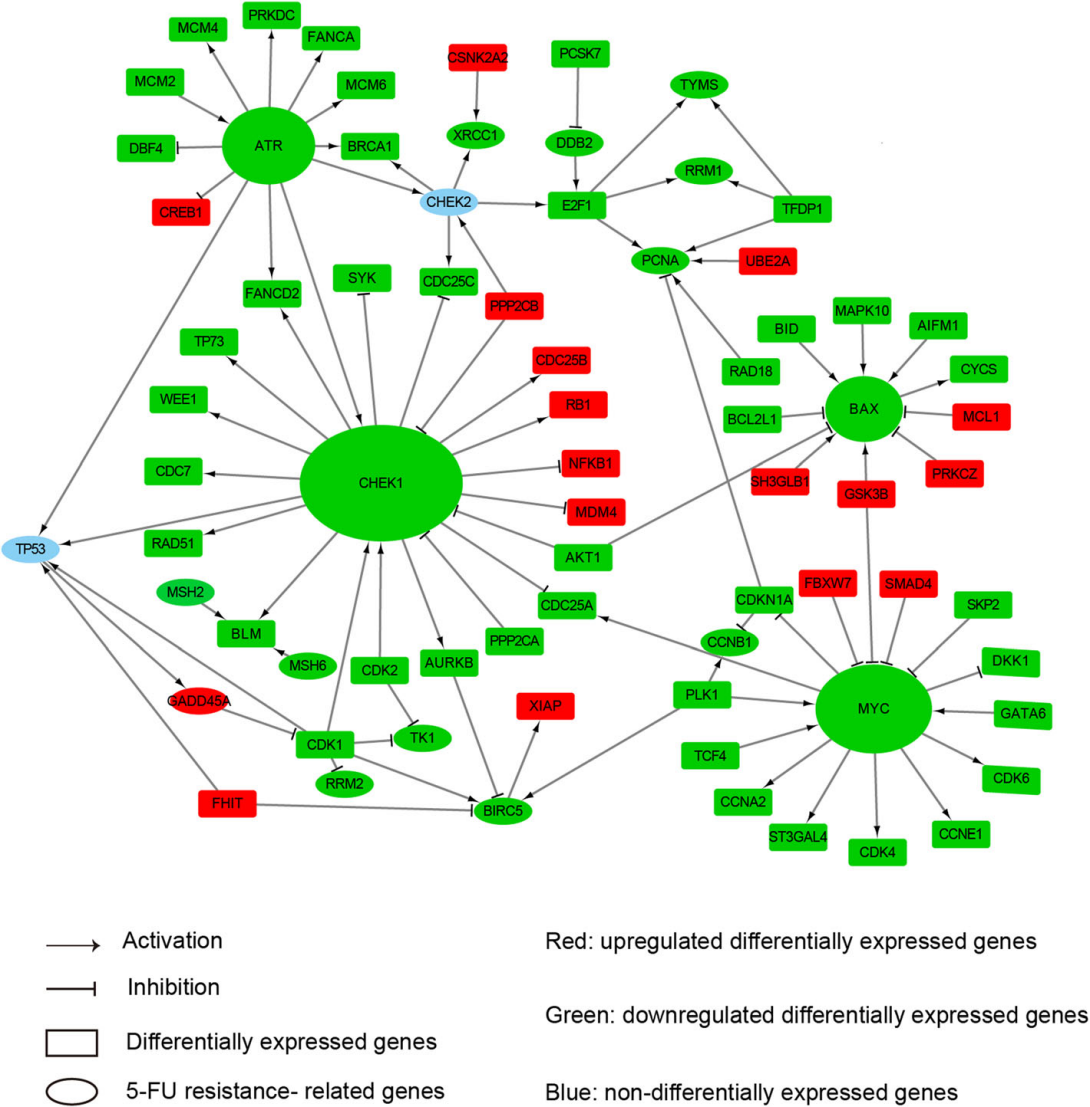
The protein-protein interaction network between DEGs of HCT-8 and 5-FU resistance-related genes in CRC. The network was consisted of DEGs of HCT-8 treated by melatonin and 5-FU resistance-related genes in CRC. The node shapes represent the types of genes. Ellipse, 5-FU resistance-related genes, of which overexpression are positively related with 5-FU resistance. Rectangle, DEGs of HCT-8 after melatonin treatment. The node colors indicate genes upregulated (Red), downregulated (Green) or non-differentially expressed (Blue) in HCT-8 after melatonin treatment

For CRC tumors, we investigated the relationship by analyzing the protein-protein interaction network. A directed regulatory network included 136 DEGs in the HCT-8 cell lines after melatonin treatment and 37 genes related with 5-FU resistance in CRC was shown in Fig. 4. Four resistance-related genes (*ATR*, *CHEK1*, *MYC* and *BAX*) were the hubs with the largest degrees in the network (all≥11), of which the expression levels were downregulated by melatonin. The *ATR*-*CHEK1* pathway is known to be responsible for DNA damage during cell cycle. It has been reported that inhibition the *ATR*-*CHEK1* pathway could enhance the efficacy of DNA damage agents in variety of carcinomas, ciplastin in CRC, gemcitabine in pancreatic cancer [45] and cytosine arabinoside in Refractory Acute Leukemias [46], and reverse the radioresistance in oral squamous cell carcinoma cells [47].

In conclusion, our results suggest that melatonin could enhance the efficacy of 5-FU in GC and CRC patients.

### Melatonin enhanced the sensitivity of CRC 5-FU resistant cells by downregulating *ATR*

**Fig. 5:**
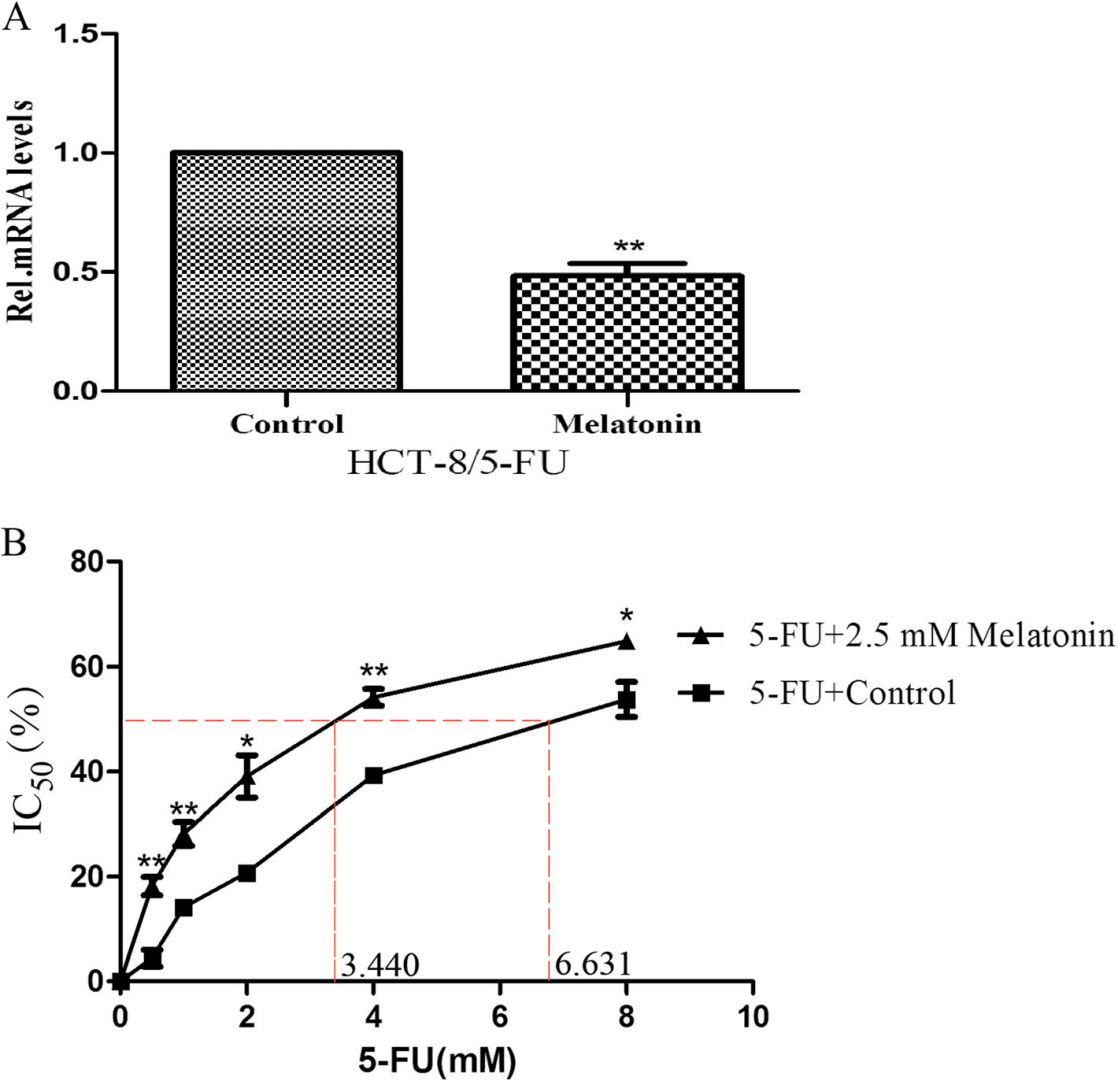
The effects of melatonin in CRC 5-FU resistant cell lines. Relative mRNA expression of *ATR* (**a**) and the IC_50_ values of 5-FU for cell viability inhibition (**b**) in HCT-8/5-FU with or without melatonin treatment for 24 h. Independent sample *t*-test was used to conduct significance analysis. * *p* < .05, ** *p* < .01, *** *p* < .001

To investigate whether melatonin downregulate the expression levels of 5-FU resistance-related genes in CRC, qRT-PCR analyses were performed. After 24 h of melatonin treatment, the expression of *ATR* in HCT-8/5-FU was significantly lower than in the control group (Fig. 5a). Then we analyzed the effect of the combined treatment of 5-FU and melatonin (2.5 mM) on the IC_50_ values for cell viability inhibition. As shown in Fig. 5b, co-treatment of 5-FU with melatonin considerably increased the sensitivity of HCT-8/5-FU to 5-FU. Compared with the cells treated with 5-FU alone, a significant reduction in the IC_50_ value of 5-FU in HCT-8/5-FU was observed (*p* < 0.01). These results showed that melatonin increased the sensitivity of CRC 5-FU resistant cells by decreasing the expression of *ATR*.

## Discussion

By performing a global analysis of gene expression profiles of four cancer cell lines across three types of gastrointestinal carcinomas, our study systematically uncovered the genes and pathways commonly altered by melatonin for the first time and confirmed its dual role in tumor cells: pro-apoptosis and anti-proliferation. Moreover, comparison of the DEG between tumor tissues and melatonin-treated cancer cell lines indicated that melatonin might exert antitumor effects through RNA transport, spliceosome and cell cycle signaling pathways. By comparing DEGs of melatonin with 5-FU-resistance related genes, we found that melatonin could downregulate the expression levels of resistance-related genes, such as *TS* in GC patients and *ATR*, *CHEK1*, *MYC* and *BAX* in CRC patients. The qRT-PCR results demonstrated the role of melatonin by decreasing expression of *ATR* to increase the sensitivity of CRC 5-FU resistant cells.

Our results showed that melatonin might downregulate the expression levels of five resistance-related genes in GC and CRC to increase the sensitivity of 5-FU, which were consistent with previous studies. Studies have established a strong association between increased *TS* expression and development of 5-FU chemoresistance. Clinical trial results have shown that *TS* expression was negatively correlated with chemotherapy response in CRC patients [48]. Recent study has shown that melatonin can abate the chemoresistance of CRC cells to 5-FU by downregulating the expression of *TS* [49]. Liang et al. revealed that downregulation of *MYC* can induce 5-FU sensitivity in nasopharyngeal carcinoma [50]. Another gene, *BAX*, which plays an important role in p53 signaling pathway, is known to induce apoptosis. It has been reported that melatonin could downregulate the expression of *MYC* and upregulate the expression of *BAX* to stimulate the apoptotic effects in breast cancer cells [51].

It is reported that melatonin can activate the MAPK cascades [41, 52, 53]. In line with these studies, the upregulated genes by melatonin in HGC-27, HCT-8 and Huh-7 cell lines were significantly enriched in the MAPK pathway with 10% FDR control. With a loosen 5% *p*-value, the upregulated genes by melatonin in the HepG2 cell lines were also enriched the pathway. Genes *MAP 3 K2*, *MAP 3 K7*, *MAP 3 K18*, *MAPK8* and *MAPK9* in the pathway, which were responsible for DNA damage or apoptosis [54], were all upregulated by melatonin treatment in four cancer cell lines. The results also supported the dual role of melatonin in tumor cells.

Melatonin has been showed to increase the efficiency of cisplatin in ovarian cancer cell lines [55], 5-FU in CRC cells [56], sorafenib in HCC cells [16, 19]. Our results indicated that melatonin may improve the chemotherapeutic effect of 5-FU in GC and CRC patients. The treatment by the combination of 5-FU and melatonin may obtain better therapeutic benefits for GC and CRC patients than 5-FU alone, which might be a good solution for patients with tumor insensitive or acquired-resistant to conventional 5-FU based chemotherapy. Therefore, in consideration of its low toxicity, it’s worth to investigate the combination of melatonin with chemotherapeutic agents in aiding cancer patients against different types of tumors. Besides, we are aware that our study is carried out in vitro and the concentration of melatonin used in this study is hardly reached in humans. The proper dose and way of melatonin administration in clinic cancer therapy need be further investigated.

## Conclusions

Our study systematically uncovered the genes and pathways commonly altered by melatonin for the first time and confirmed its dual role in tumor cells: pro-apoptosis and anti-proliferation. Our results further indicated that melatonin might increase the sensitivity of 5-FU in GC and CRC.

## Supplementary information

**Additional file 1: Supplementary Table S1.** The sequence of the target gene primers. **Supplementary Table S2.** DEGs detected in four cancer cell lines by Student’s *t*-test and the reproducibility-based PD. **Supplementary Table S3.** The pathways enriched by the upregulated DEGs of four cancer cell lines treated by melatonin. *p* (< 0.1) was adjusted by Benjamini and Hochberg. **Supplementary Table S4.** The pathways enriched by the downregulated DEGs of four cancer cell lines treated by melatonin. *p* (< 0.1) was adjusted by Benjamini and Hochberg. **Supplementary Table S5.** Pathways enriched by genes upregulated in tumor tissues but downregulated in cell lines after melatonin treatment. *p* (< 0.1) was adjusted by Benjamini and Hochberg. **Supplementary Table S6.** Pathways enriched by genes downregulated in tumor tissues but upregulated in cell lines after melatonin treatment. *p* (< 0.1) was adjusted by Benjamini and Hochberg. **Supplementary Table S7.** Pathways enriched by genes upregulated in the resistance high-risk GC patients but downregulated in the HGC-27 cell lines treated by melatonin. *p* (< 0.1) was adjusted by Benjamini and Hochberg.

## Data Availability

Expression profiling data measured in our study are available in the Gene Expression Omnibus repository (GEO accession number: GSE132119).

## Abbreviations

GC: gastric carcinoma
CRC: colorectal carcinoma
HCC: hepatocellular carcinoma
5-FU: 5-fluorouracil
DEGs: differentially expressed genes
FDR: False Discovery Rate

## References

[1] Dominguez-Rodriguez A, Abreu-Gonzalez P, Sanchez-Sanchez JJ, Kaski JC, Reiter RJ (2010). Melatonin and circadian biology in human cardiovascular disease. J Pineal Res.

[2] Garcia JJ, Lopez-Pingarron L, Almeida-Souza P, Tres A, Escudero P, Garcia-Gil FA, Tan DX, Reiter RJ, Ramirez JM, Bernal-Perez M (2014). Protective effects of melatonin in reducing oxidative stress and in preserving the fluidity of biological membranes: a review. J Pineal Res.

[3] Tan DX, Manchester LC, Hardeland R, Lopez-Burillo S, Mayo JC, Sainz RM, Reiter RJ (2003). Melatonin: a hormone, a tissue factor, an autocoid, a paracoid, and an antioxidant vitamin. J Pineal Res.

[4] Volt H, Garcia JA, Doerrier C, Diaz-Casado ME, Guerra-Librero A, Lopez LC, Escames G, Tresguerres JA, Acuna-Castroviejo D (2016). Same molecule but different expression: aging and sepsis trigger NLRP3 inflammasome activation, a target of melatonin. J Pineal Res.

[5] Santello FH, Frare EO, Caetano LC, AlonsoToldo MP, Do Prado JC, Jr.: Melatonin enhances pro-inflammatory cytokine levels and protects against Chagas disease. J Pineal Res 2008, 45(1):79–85. 10.1111/j.1600-079X.2008.00558.x 18284549

[6] Konturek SJ, Konturek PC, Brzozowska I, Pawlik M, Sliwowski Z, Czesnikiewicz-Guzik M, Kwiecien S, Brzozowski T, Bubenik GA, Pawlik WW (2007). Localization and biological activities of melatonin in intact and diseased gastrointestinal tract (GIT). J Physiol Pharmacol.

[7] Bubenik GA (2008). Thirty four years since the discovery of gastrointestinal melatonin. J Physiol Pharmacol.

[8] Lozanovski VJ, Polychronidis G, Gross W, Gharabaghi N, Mehrabi A, Hackert T, Schemmer P, Herr I (2020). Broccoli sprout supplementation in patients with advanced pancreatic cancer is difficult despite positive effects-results from the POUDER pilot study. Investig New Drugs.

[9] Maneikyte J, Bausys A, Leber B, Horvath A, Feldbacher N, Hoefler G, Strupas K, Stiegler P, Schemmer P (2019). Dietary glycine decreases both tumor volume and vascularization in a combined colorectal liver metastasis and chemotherapy model. Int J Biol Sci.

[10] Martin-Renedo J, Mauriz JL, Jorquera F, Ruiz-Andres O, Gonzalez P, Gonzalez-Gallego J (2008). Melatonin induces cell cycle arrest and apoptosis in hepatocarcinoma HepG2 cell line. J Pineal Res.

[11] Carbajo-Pescador S, Garcia-Palomo A, Martin-Renedo J, Piva M, Gonzalez-Gallego J, Mauriz JL (2011). Melatonin modulation of intracellular signaling pathways in hepatocarcinoma HepG2 cell line: role of the MT1 receptor. J Pineal Res.

[12] Farriol M, Venereo Y, Orta X, Castellanos JM, Segovia-Silvestre T (2000). In vitro effects of melatonin on cell proliferation in a colon adenocarcinoma line. J Applied Toxicol.

[13] Hill SM, Blask DE (1988). Effects of the pineal hormone melatonin on the proliferation and morphological characteristics of human breast cancer cells (MCF-7) in culture. Cancer Res.

[14] Petranka J, Baldwin W, Biermann J, Jayadev S, Barrett JC, Murphy E (1999). The oncostatic action of melatonin in an ovarian carcinoma cell line. J Pineal Res.

[15] Carbajo-Pescador S, Martin-Renedo J, Garcia-Palomo A, Tunon MJ, Mauriz JL, Gonzalez-Gallego J (2009). Changes in the expression of melatonin receptors induced by melatonin treatment in hepatocarcinoma HepG2 cells. J Pineal Res.

[16] Prieto-Dominguez N, Ordonez R, Fernandez A, Mendez-Blanco C, Baulies A, Garcia-Ruiz C, Fernandez-Checa JC, Mauriz JL, Gonzalez-Gallego J (2016). Melatonin-induced increase in sensitivity of human hepatocellular carcinoma cells to sorafenib is associated with reactive oxygen species production and mitophagy. J Pineal Res.

[17] Lissoni P, Paolorossi F, Tancini G, Ardizzoia A, Barni S, Brivio F, Maestroni GJ, Chilelli M (1996). A phase II study of tamoxifen plus melatonin in metastatic solid tumour patients. Br J Cancer.

[18] Pariente R, Pariente JA, Rodriguez AB, Espino J (2016). Melatonin sensitizes human cervical cancer HeLa cells to cisplatin-induced cytotoxicity and apoptosis: effects on oxidative stress and DNA fragmentation. J Pineal Res.

[19] Lin S, Hoffmann K, Gao C, Petrulionis M, Herr I, Schemmer P. Melatonin promotes sorafenib-induced apoptosis through synergistic activation of JNK/c-jun pathway in human hepatocellular carcinoma. J Pineal Res. 2017;62(3). 10.1111/jpi.12398. 10.1111/jpi.12398 28178378

[20] Hara M, Yoshida M, Nishijima H, Yokosuka M, Iigo M, Ohtani-Kaneko R, Shimada A, Hasegawa T, Akama Y, Hirata K (2001). Melatonin, a pineal secretory product with antioxidant properties, protects against cisplatin-induced nephrotoxicity in rats. J Pineal Res.

[21] Lopez-Gonzalez MA, Guerrero JM, Rojas F, Delgado F (2000). Ototoxicity caused by cisplatin is ameliorated by melatonin and other antioxidants. J Pineal Res.

[22] Lissoni P, Paolorossi F, Ardizzoia A, Barni S, Chilelli M, Mancuso M, Tancini G, Conti A, Maestroni GJ (1997). A randomized study of chemotherapy with cisplatin plus etoposide versus chemoendocrine therapy with cisplatin, etoposide and the pineal hormone melatonin as a first-line treatment of advanced non-small cell lung cancer patients in a poor clinical state. J Pineal Res.

[23] Lissoni P, Barni S, Mandala M, Ardizzoia A, Paolorossi F, Vaghi M, Longarini R, Malugani F, Tancini G (1999). Decreased toxicity and increased efficacy of cancer chemotherapy using the pineal hormone melatonin in metastatic solid tumour patients with poor clinical status. Eur J Cancer.

[24] Cui J, Chen Y, Chou WC, Sun L, Chen L, Suo J, Ni Z, Zhang M, Kong X, Hoffman LL (2011). An integrated transcriptomic and computational analysis for biomarker identification in gastric cancer. Nucleic Acids Res.

[25] Zhang X, Ni Z, Duan Z, Xin Z, Wang H, Tan J, Wang G, Li F (2015). Overexpression of E2F mRNAs associated with gastric cancer progression identified by the transcription factor and miRNA co-regulatory network analysis. PLoS One.

[26] Sabates-Bellver J, Van der Flier LG, de Palo M, Cattaneo E, Maake C, Rehrauer H, Laczko E, Kurowski MA, Bujnicki JM, Menigatti M (2007). Transcriptome profile of human colorectal adenomas. Molecular Cancer Res.

[27] Uddin S, Ahmed M, Hussain A, Abubaker J, Al-Sanea N, AbdulJabbar A, Ashari LH, Alhomoud S, Al-Dayel F, Jehan Z (2011). Genome-wide expression analysis of middle eastern colorectal cancer reveals FOXM1 as a novel target for cancer therapy. Am J Pathol.

[28] Roessler S, Jia HL, Budhu A, Forgues M, Ye QH, Lee JS, Thorgeirsson SS, Sun Z, Tang ZY, Qin LX (2010). A unique metastasis gene signature enables prediction of tumor relapse in early-stage hepatocellular carcinoma patients. Cancer Res.

[29] Kim JH, Sohn BH, Lee HS, Kim SB, Yoo JE, Park YY, Jeong W, Lee SS, Park ES, Kaseb A (2014). Genomic predictors for recurrence patterns of hepatocellular carcinoma: model derivation and validation. PLoS Med.

[30] Irizarry RA, Hobbs B, Collin F, Beazer-Barclay YD, Antonellis KJ, Scherf U, Speed TP (2003). Exploration, normalization, and summaries of high density oligonucleotide array probe level data. Biostatistics.

[31] Ao L, Yan H, Zheng T, Wang H, Tong M, Guan Q, Li X, Cai H, Li M, Guo Z (2015). Identification of reproducible drug-resistance-related dysregulated genes in small-scale cancer cell line experiments. Sci Rep.

[32] Huang H, Li X, Guo Y, Zhang Y, Deng X, Chen L, Zhang J, Guo Z, Ao L (2016). Identifying reproducible cancer-associated highly expressed genes with important functional significances using multiple datasets. Sci Rep.

[33] Soong R, Diasio RB (2005). Advances and challenges in fluoropyrimidine pharmacogenomics and pharmacogenetics. Pharmacogenomics.

[34] Tan WL, Bhattacharya B, Loh M, Balasubramanian I, Akram M, Dong D, Wong L, Thakkar B, Salto-Tellez M, Soo RA (2011). Low cytosine triphosphate synthase 2 expression renders resistance to 5-fluorouracil in colorectal cancer. Cancer Biol Ther.

[35] Lo Surdo P, Calderone A, Cesareni G, Perfetto L: SIGNOR: A Database of Causal Relationships Between Biological Entities-A Short Guide to Searching and Browsing. Curr Protocols Bioinformatics 2017, 58:8 23 21–28 23 16. 10.1002/cpbi.28 28654729

[36] Kanehisa M, Goto S (2000). KEGG: Kyoto encyclopedia of genes and genomes. Nucleic Acids Res.

[37] Belanger BF, Williams WJ, Yin TC (1976). A flexible renewal process simulator for neural spike trains. IEEE Trans Biomed Eng.

[38] Colombo J, Maciel JM, Ferreira LC, RF DAS, Zuccari DA: Effects of melatonin on HIF-1alpha and VEGF expression and on the invasive properties of hepatocarcinoma cells. Oncol Lett 2016, 12(1):231–237. 10.3892/ol.2016.4605 PMC4907066 27347130

[39] Prieto-Dominguez N, Mendez-Blanco C, Carbajo-Pescador S, Fondevila F, Garcia-Palomo A, Gonzalez-Gallego J, Mauriz JL (2017). Melatonin enhances sorafenib actions in human hepatocarcinoma cells by inhibiting mTORC1/p70S6K/HIF-1alpha and hypoxia-mediated mitophagy. Oncotarget.

[40] Carbajo-Pescador S, Steinmetz C, Kashyap A, Lorenz S, Mauriz JL, Heise M, Galle PR, Gonzalez-Gallego J, Strand S (2013). Melatonin induces transcriptional regulation of Bim by FoxO3a in HepG2 cells. Br J Cancer.

[41] Mediavilla MD, Sanchez-Barcelo EJ, Tan DX, Manchester L, Reiter RJ (2010). Basic mechanisms involved in the anti-cancer effects of melatonin. Curr Med Chem.

[42] Li X, Cai H, Zheng W, Tong M, Li H, Ao L, Li J, Hong G, Li M, Guan Q (2016). An individualized prognostic signature for gastric cancer patients treated with 5-fluorouracil-based chemotherapy and distinct multi-omics characteristics of prognostic groups. Oncotarget.

[43] Longley DB, Harkin DP, Johnston PG (2003). 5-fluorouracil: mechanisms of action and clinical strategies. Nat Rev Cancer.

[44] Zembutsu H, Ohnishi Y, Tsunoda T, Furukawa Y, Katagiri T, Ueyama Y, Tamaoki N, Nomura T, Kitahara O, Yanagawa R (2002). Genome-wide cDNA microarray screening to correlate gene expression profiles with sensitivity of 85 human cancer xenografts to anticancer drugs. Cancer Res.

[45] Venkatesha VA, Parsels LA, Parsels JD, Zhao L, Zabludoff SD, Simeone DM, Maybaum J, Lawrence TS, Morgan MA (2012). Sensitization of pancreatic cancer stem cells to gemcitabine by Chk1 inhibition. Neoplasia.

[46] Karp JE, Thomas BM, Greer JM, Sorge C, Gore SD, Pratz KW, Smith BD, Flatten KS, Peterson K, Schneider P (2012). Phase I and pharmacologic trial of cytosine arabinoside with the selective checkpoint 1 inhibitor Sch 900776 in refractory acute leukemias. Clin Cancer Res.

[47] Sankunny M, Parikh RA, Lewis DW, Gooding WE, Saunders WS, Gollin SM (2014). Targeted inhibition of ATR or CHEK1 reverses radioresistance in oral squamous cell carcinoma cells with distal chromosome arm 11q loss. Genes Chromosomes Cancer.

[48] Lenz HJ, Hayashi K, Salonga D, Danenberg KD, Danenberg PV, Metzger R, Banerjee D, Bertino JR, Groshen S, Leichman LP (1998). p53 point mutations and thymidylate synthase messenger RNA levels in disseminated colorectal cancer: an analysis of response and survival. Clin Cancer Res.

[49] Sakatani A, Sonohara F, Goel A (2019). Melatonin-mediated downregulation of thymidylate synthase as a novel mechanism for overcoming 5-fluorouracil associated chemoresistance in colorectal cancer cells. Carcinogenesis.

[50] Liang Z, Liu Z, Cheng C, Wang H, Deng X, Liu J, Liu C, Li Y, Fang W (2019). VPS33B interacts with NESG1 to modulate EGFR/PI3K/AKT/c-Myc/P53/miR-133a-3p signaling and induce 5-fluorouracil sensitivity in nasopharyngeal carcinoma. Cell Death Dis.

[51] Alonso-Gonzalez C, Menendez-Menendez J, Gonzalez-Gonzalez A, Gonzalez A, Cos S, Martinez-Campa C (2018). Melatonin enhances the apoptotic effects and modulates the changes in gene expression induced by docetaxel in MCF7 human breast cancer cells. Int J Oncol.

[52] Chan AS, Lai FP, Lo RK, Voyno-Yasenetskaya TA, Stanbridge EJ, Wong YH (2002). Melatonin mt1 and MT2 receptors stimulate c-Jun N-terminal kinase via pertussis toxin-sensitive and -insensitive G proteins. Cell Signal.

[53] Hazlerigg DG, Thompson M, Hastings MH, Morgan PJ (1996). Regulation of mitogen-activated protein kinase in the pars tuberalis of the ovine pituitary: interactions between melatonin, insulin-like growth factor-1, and forskolin. Endocrinology.

[54] Raman M, Chen W, Cobb MH (2007). Differential regulation and properties of MAPKs. Oncogene.

[55] Futagami M, Sato S, Sakamoto T, Yokoyama Y, Saito Y (2001). Effects of melatonin on the proliferation and cis-diamminedichloroplatinum (CDDP) sensitivity of cultured human ovarian cancer cells. Gynecol Oncol.

[56] Gao Y, Xiao X, Zhang C, Yu W, Guo W, Zhang Z, Li Z, Feng X, Hao J, Zhang K et al. Melatonin synergizes the chemotherapeutic effect of 5-fluorouracil in colon cancer by suppressing PI3K/AKT and NF-κB/iNOS signaling pathways. J Pineal Res. 2017;62(2). 10.1111/jpi.12380. 10.1111/jpi.12380 27865009

