# Supplementary material for: Transcriptomic analysis on the effects of melatonin in gastrointestinal carcinomas
Source: BMC Gastroenterol. 2020 Jul 20;20:233. doi: 10.1186/s12876-020-01383-z (PMC7372748; doi:10.1186/s12876-020-01383-z)
Supplement: Supplementary file 1 — Additional file 1: Supplementary Table S1. The sequence of the target gene primers. Supplementary Table S2. DEGs detected in four cancer cell lines by Student’s t-test and the reproducibility-based PD. Supplementary Table S3. The pathways enriched by the upregulated DEGs of four cancer cell lines treated by melatonin. p (< 0.1) was adjusted by Benjamini and Hochberg. Supplementary Table S4. The pathways enriched by the downregulated DEGs of four cancer cell lines treated by melatonin. p (< 0.1) was adjusted by Benjamini and Hochberg. Supplementary Table S5. Pathways enriched by genes upregulated in tumor tissues but downregulated in cell lines after melatonin treatment. p (< 0.1) was adjusted by Benjamini and Hochberg. Supplementary Table S6. Pathways enriched by genes downregulated in tumor tissues but upregulated in cell lines after melatonin treatment. p (< 0.1) was adjusted by Benjamini and Hochberg. Supplementary Table S7. Pathways enriched by genes upregulated in the resistance high-risk GC patients but downregulated in the HGC-27 cell lines treated by melatonin. p (< 0.1) was adjusted by Benjamini and Hochberg. [file 12876_2020_1383_MOESM1_ESM.docx]

**Supplementary Table S1**. **The sequence of the target gene primers**

| primers | sequence |
| --- | --- |
| *GAPDH* | F：5’-TGCACCACCAACTGCTTAGC |
|  | R：5’-AGCTCAGGGATGACCTTGCC |
| *ATR* | F：5’-GGCCAAAGGCAGTTGTATTGA |
|  | R：5’-GTGAGTACCCCAAAAATAGCAGG |

**Supplementary Table S2.** **DEGs detected in four cancer cell lines by Student's *t*-test and the reproducibility-based PD**

|  |  | DEGs | common | consistent | opposite | union | up | down |
| --- | --- | --- | --- | --- | --- | --- | --- | --- |
| HGC-27 | T | 6236 | 5265 | 5265 | 0 | 7898 | 4114 | 3784 |
|  | PD | 7287 |  |  |  |  |  |  |
| HCT-8 | T | 4358 | 3679 | 3679 | 0 | 6363 | 3242 | 3121 |
|  | PD | 5684 |  |  |  |  |  |  |
| Huh-7 | T | 8321 | 7469 | 7469 | 0 | 10282 | 4673 | 5609 |
|  | PD | 9610 |  |  |  |  |  |  |
| HepG2 | T | 6096 | 5414 | 5414 | 0 | 7815 | 3837 | 3978 |
|  | PD | 7133 |  |  |  |  |  |  |

**Supplementary Table S3. The pathways enriched by the upregulated DEGs of four cancer cell lines treated by melatonin. p(<0.1) was adjusted by Benjamini and Hochberg.**

| Cell line | Pathway Name | *p* |
| --- | --- | --- |
| HGC-27 | 'Other glycan degradation ' | 0.05875309 |
|  | 'Glycosaminoglycan biosynthesis - chondroitin sulfate / dermatan sulfate ' | 0.05548903 |
|  | 'Inositol phosphate metabolism ' | 4.84E-05 |
|  | 'Sphingolipid metabolism ' | 0.00544435 |
|  | 'MAPK signaling pathway ' | 0.09556705 |
|  | 'ErbB signaling pathway ' | 0.00024394 |
|  | 'FoxO signaling pathway ' | 0.00325878 |
|  | 'Phosphatidylinositol signaling system ' | 1.27E-06 |
|  | 'SNARE interactions in vesicular transport ' | 0.02384376 |
|  | 'Regulation of autophagy ' | 0.00293848 |
|  | 'Protein processing in endoplasmic reticulum ' | 0.00028771 |
|  | 'Lysosome ' | 9.86E-05 |
|  | 'Endocytosis ' | 1.55E-06 |
|  | 'Osteoclast differentiation ' | 0.08196815 |
|  | 'Jak-STAT signaling pathway ' | 0.0606204 |
|  | 'Fc epsilon RI signaling pathway ' | 0.07880694 |
|  | 'Neurotrophin signaling pathway ' | 0.00214869 |
|  | 'Inflammatory mediator regulation of TRP channels ' | 0.06566547 |
|  | 'Insulin signaling pathway ' | 0.00013191 |
|  | 'GnRH signaling pathway ' | 0.0230124 |
|  | 'Prolactin signaling pathway ' | 0.02080151 |
|  | 'Thyroid hormone signaling pathway ' | 0.08242505 |
|  | 'Mineral absorption ' | 0.05071964 |
| HCT-8 | 'Glycosaminoglycan biosynthesis - chondroitin sulfate / dermatan sulfate' | 0.05353849 |
|  | 'Glycosaminoglycan biosynthesis - heparan sulfate / heparin' | 0.02157806 |
|  | 'Inositol phosphate metabolism' | 0.0025311 |
|  | 'Sphingolipid metabolism' | 0.03449741 |
|  | 'Basal transcription factors' | 0.08068405 |
|  | 'MAPK signaling pathway' | 0.00338658 |
|  | 'ErbB signaling pathway' | 0.03089969 |
|  | 'Ras signaling pathway' | 0.06830248 |
|  | 'cGMP-PKG signaling pathway' | 0.03190322 |
|  | 'cAMP signaling pathway' | 0.07816338 |
|  | 'Chemokine signaling pathway' | 0.01176788 |
|  | 'FoxO signaling pathway' | 0.0036014 |
|  | 'Phosphatidylinositol signaling system' | 0.00128543 |
|  | 'Sphingolipid signaling pathway' | 0.05000505 |
|  | 'Ubiquitin mediated proteolysis' | 0.02766539 |
|  | 'SNARE interactions in vesicular transport' | 0.0700473 |
|  | 'Regulation of autophagy' | 0.00375012 |
|  | 'Protein processing in endoplasmic reticulum' | 6.19E-06 |
|  | 'Lysosome' | 0.00171637 |
|  | 'Endocytosis' | 1.87E-06 |
|  | 'Phagosome' | 0.07291257 |
|  | 'AMPK signaling pathway' | 0.07076808 |
|  | 'Wnt signaling pathway' | 0.016728 |
|  | 'Osteoclast differentiation' | 0.03136942 |
|  | 'Hippo signaling pathway' | 0.04821915 |
|  | 'Adherens junction' | 0.07469145 |
|  | 'Antigen processing and presentation' | 0.08103077 |
|  | 'Toll-like receptor signaling pathway' | 0.03257264 |
|  | 'NOD-like receptor signaling pathway' | 0.06663794 |
|  | 'RIG-I-like receptor signaling pathway' | 0.02878966 |
|  | 'T cell receptor signaling pathway' | 0.09400748 |
|  | 'TNF signaling pathway' | 0.02943643 |
|  | 'Circadian rhythm' | 0.0989413 |
|  | 'Neurotrophin signaling pathway' | 0.07161387 |
|  | 'Glutamatergic synapse' | 0.03277254 |
|  | 'Long-term depression' | 0.03487783 |
|  | 'Insulin signaling pathway' | 0.015334 |
|  | 'GnRH signaling pathway' | 0.03485377 |
|  | 'Estrogen signaling pathway' | 0.05507555 |
|  | 'Melanogenesis' | 0.01760581 |
|  | 'Prolactin signaling pathway' | 0.079005 |
|  | 'Thyroid hormone synthesis' | 0.0398135 |
|  | 'Glucagon signaling pathway' | 0.03893225 |
|  | 'Gastric acid secretion' | 0.06549484 |
| Huh-7 | 'Other glycan degradation' | 0.06617349 |
|  | 'Glycosaminoglycan biosynthesis - chondroitin sulfate / dermatan sulfate' | 0.05674518 |
|  | 'Glycosaminoglycan biosynthesis - heparan sulfate / heparin' | 0.09928753 |
|  | 'Inositol phosphate metabolism' | 0.00100487 |
|  | 'Glycosylphosphatidylinositol(GPI)-anchor biosynthesis' | 0.01436803 |
|  | 'Sphingolipid metabolism' | 0.02324946 |
|  | 'ErbB signaling pathway' | 0.0047336 |
|  | 'Ras signaling pathway' | 0.05191487 |
|  | 'FoxO signaling pathway' | 0.000406 |
|  | 'Phosphatidylinositol signaling system' | 0.00035394 |
|  | 'Sphingolipid signaling pathway' | 0.05913633 |
|  | 'Regulation of autophagy' | 0.02547501 |
|  | 'Protein processing in endoplasmic reticulum' | 0.04259869 |
|  | 'Lysosome' | 3.10E-09 |
|  | 'Endocytosis' | 0.00016098 |
|  | 'Peroxisome' | 0.07511861 |
|  | 'mTOR signaling pathway' | 0.00492056 |
|  | 'Focal adhesion' | 0.09961366 |
|  | 'Signaling pathways regulating pluripotency of stem cells' | 2.36E-02 |
|  | 'Natural killer cell mediated cytotoxicity' | 0.09526682 |
|  | 'T cell receptor signaling pathway' | 0.07579144 |
|  | 'Synaptic vesicle cycle' | 0.01111334 |
|  | 'Neurotrophin signaling pathway' | 0.02061751 |
|  | 'Insulin signaling pathway' | 0.00019734 |
|  | 'Estrogen signaling pathway' | 0.02371173 |
|  | 'Prolactin signaling pathway' | 0.09989585 |
|  | 'Collecting duct acid secretion' | 0.0744772 |
| HepG2 | 'Other glycan degradation' | 0.03931713 |
|  | 'Mucin type O-Glycan biosynthesis' | 0.02174266 |
|  | 'Other types of O-glycan biosynthesis' | 0.06578796 |
|  | 'Glycosaminoglycan degradation' | 0.02075436 |
|  | 'Glycosaminoglycan biosynthesis - chondroitin sulfate / dermatan sulfate' | 0.01837388 |
|  | 'Glycosaminoglycan biosynthesis - heparan sulfate / heparin' | 0.03040757 |
|  | 'Inositol phosphate metabolism' | 0.00814298 |
|  | 'Sphingolipid metabolism' | 0.00089962 |
|  | 'Glycosphingolipid biosynthesis - ganglio series' | 0.01952778 |
|  | 'ErbB signaling pathway' | 0.03134735 |
|  | 'cGMP-PKG signaling pathway' | 0.02290542 |
|  | 'cAMP signaling pathway' | 0.01168822 |
|  | 'Chemokine signaling pathway' | 0.09593168 |
|  | 'FoxO signaling pathway' | 0.03293275 |
|  | 'Phosphatidylinositol signaling system' | 0.00074653 |
|  | 'Sphingolipid signaling pathway' | 0.00701529 |
|  | 'SNARE interactions in vesicular transport' | 0.09418292 |
|  | 'Protein processing in endoplasmic reticulum' | 0.01301264 |
|  | 'Lysosome' | 2.92E-08 |
|  | 'Endocytosis' | 0.00034773 |
|  | 'mTOR signaling pathway' | 0.07234826 |
|  | 'AMPK signaling pathway' | 0.00941332 |
|  | 'Wnt signaling pathway' | 0.02559694 |
|  | 'Notch signaling pathway' | 0.02223648 |
|  | 'Adherens junction' | 0.06669565 |
|  | 'Fc epsilon RI signaling pathway' | 0.03250963 |
|  | 'Fc gamma R-mediated phagocytosis' | 0.02380647 |
|  | 'Long-term potentiation' | 0.00715704 |
|  | 'Synaptic vesicle cycle' | 0.06292969 |
|  | 'Neurotrophin signaling pathway' | 0.01996221 |
|  | 'Dopaminergic synapse' | 0.03236435 |
|  | 'Inflammatory mediator regulation of TRP channels' | 0.02160634 |
|  | 'Insulin signaling pathway' | 0.00037368 |
|  | 'Insulin secretion' | 0.09466482 |
|  | 'GnRH signaling pathway' | 0.01604228 |
|  | 'Estrogen signaling pathway' | 0.0149728 |
|  | 'Melanogenesis' | 0.01617543 |
|  | 'Thyroid hormone signaling pathway' | 0.05241634 |
|  | 'Glucagon signaling pathway' | 0.01997241 |
|  | 'Endocrine and other factor-regulated calcium reabsorption' | 0.017135 |
|  | 'Collecting duct acid secretion' | 0.09898246 |
|  | 'Gastric acid secretion' | 0.03271055 |

**Supplementary Table S4. The pathways enriched by the downregulated DEGs of four cancer cell lines treated by melatonin. p(<0.1) was adjusted by Benjamini and Hochberg.**

| Cell line | Pathway Name | *p* |
| --- | --- | --- |
| HGC-27 | 'Glycolysis / Gluconeogenesis' | 0.0830709 |
|  | 'Purine metabolism' | 0.0315894 |
|  | 'Pyrimidine metabolism' | 0.0231126 |
|  | 'Ribosome biogenesis in eukaryotes' | 0.0008305 |
|  | 'Ribosome' | 0.0021264 |
|  | 'RNA transport' | 6.60E-05 |
|  | 'DNA replication' | 0.0841548 |
|  | 'Spliceosome' | 6.76E-12 |
|  | 'Proteasome' | 0.001019 |
|  | 'Cell cycle' | 0.0009029 |
| HCT-8 | 'Glycolysis / Gluconeogenesis' | 0.0421939 |
|  | 'Oxidative phosphorylation' | 0.0254568 |
|  | 'Purine metabolism' | 0.0786713 |
|  | 'Pyrimidine metabolism' | 0.0032994 |
|  | 'Ribosome biogenesis in eukaryotes' | 0.003055 |
|  | 'Ribosome' | 0.0031627 |
|  | 'RNA transport' | 0.0004655 |
|  | 'DNA replication' | 7.45E-05 |
|  | 'Spliceosome' | 8.30E-07 |
|  | 'Proteasome' | 0.0032295 |
|  | 'Mismatch repair' | 0.0094959 |
|  | 'Neuroactive ligand-receptor interaction' | 0.0039683 |
|  | 'Cell cycle' | 0.0035579 |
|  | 'Olfactory transduction' | 0.0054189 |
| Huh-7 | 'Glycolysis / Gluconeogenesis' | 0.0415466 |
|  | 'Pyrimidine metabolism' | 0.0160763 |
|  | 'Biosynthesis of antibiotics' | 0.0325075 |
|  | 'Ribosome biogenesis in eukaryotes' | 2.17E-05 |
|  | 'RNA transport' | 0.0155267 |
|  | 'DNA replication' | 0.0202405 |
|  | 'Spliceosome' | 5.83E-07 |
|  | 'Proteasome' | 0.0001237 |
|  | 'Neuroactive ligand-receptor interaction' | 0.0198703 |
|  | 'Cell cycle' | 0.0156697 |
|  | 'Olfactory transduction' | 3.35E-07 |
| HepG2 | 'Pyrimidine metabolism' | 0.0057712 |
|  | 'Aminoacyl-tRNA biosynthesis' | 0.0137343 |
|  | 'Biosynthesis of antibiotics' | 0.044103 |
|  | 'Carbon metabolism' | 0.0961195 |
|  | 'Biosynthesis of amino acids' | 0.0804994 |
|  | 'Ribosome biogenesis in eukaryotes' | 0.0115841 |
|  | 'RNA transport' | 0.0064498 |
|  | 'DNA replication' | 0.041395 |
|  | 'Spliceosome' | 0.0132802 |
|  | 'Proteasome' | 0.0079218 |
|  | 'Cell cycle' | 0.0846878 |
|  | 'Olfactory transduction' | 0.0383943 |

**Supplementary Table S5.** **Pathways enriched by genes upregulated in tumor tissues but downregulated in cell lines after melatonin treatment. p(<0.1) was adjusted by Benjamini and Hochberg.**

| Cell line | Pathway Name | *p* |
| --- | --- | --- |
| HGC-27 | 'Pyrimidine metabolism' | 0.0643046 |
|  | 'Ribosome biogenesis in eukaryotes' | 0.00022 |
|  | 'RNA transport' | 0.0241605 |
|  | 'Spliceosome' | 0.0002081 |
|  | 'Cell cycle' | 0.0008885 |
| HCT-8 | 'Pyrimidine metabolism' | 0.0203541 |
|  | 'Ribosome biogenesis in eukaryotes' | 0.000209 |
|  | 'RNA transport' | 0.0050073 |
|  | 'DNA replication' | 0.007058 |
|  | 'Spliceosome' | 0.0189457 |
|  | 'Fanconi anemia pathway' | 0.0162705 |
|  | 'Cell cycle' | 1.25E-06 |
| Huh-7 | 'Ribosome biogenesis in eukaryotes' | 0.0003492 |
|  | 'RNA transport' | 1.26E-05 |
|  | 'RNA degradation' | 0.0089247 |
|  | 'DNA replication' | 1.21E-05 |
|  | 'Spliceosome' | 8.94E-12 |
|  | 'Proteasome' | 0.001578 |
|  | 'Base excision repair' | 0.0490602 |
|  | 'Mismatch repair' | 0.0264065 |
|  | 'Cell cycle' | 1.98E-06 |
|  | 'Oocyte meiosis' | 0.0008842 |
| HepG2 | 'Aminoacyl-tRNA biosynthesis' | 0.0118264 |
|  | 'Ribosome biogenesis in eukaryotes' | 0.0013134 |
|  | 'RNA transport' | 7.12E-05 |
|  | 'RNA degradation' | 0.0614308 |
|  | 'DNA replication' | 7.99E-05 |
|  | 'Spliceosome' | 3.53E-06 |
|  | 'Proteasome' | 0.0101369 |
|  | 'Base excision repair' | 0.0554308 |
|  | 'Cell cycle' | 0.0027603 |

**Supplementary Table S6.** **Pathways enriched by genes downregulated in tumor tissues but upregulated in cell lines after melatonin treatment. p(<0.1) was adjusted by Benjamini and Hochberg.**

| Cell line | Pathway Name | *p* |
| --- | --- | --- |
| HGC-27 | 'Inositol phosphate metabolism' | 0.042462 |
|  | 'MAPK signaling pathway' | 0.0536996 |
|  | 'Calcium signaling pathway' | 0.0524699 |
|  | 'Phosphatidylinositol signaling system' | 0.0529823 |
|  | 'Regulation of autophagy' | 0.0465638 |
|  | 'Adrenergic signaling in cardiomyocytes' | 0.0992758 |
|  | 'Cholinergic synapse' | 0.0565782 |
|  | 'Insulin signaling pathway' | 0.0697811 |
|  | 'Oxytocin signaling pathway' | 0.0653106 |
| HCT-8 | 'Inositol phosphate metabolism' | 0.0044689 |
|  | 'MAPK signaling pathway' | 0.0272479 |
|  | 'Calcium signaling pathway' | 0.0054907 |
|  | 'cGMP-PKG signaling pathway' | 0.0045098 |
|  | 'cAMP signaling pathway' | 0.0174307 |
|  | 'Phosphatidylinositol signaling system' | 0.0002647 |
|  | 'Sphingolipid signaling pathway' | 0.0764555 |
|  | 'Regulation of autophagy' | 0.0740102 |
|  | 'Protein processing in endoplasmic reticulum' | 0.0688118 |
|  | 'Lysosome' | 0.065684 |
|  | 'Endocytosis' | 0.0501902 |
|  | 'Adrenergic signaling in cardiomyocytes' | 0.0262249 |
|  | 'Vascular smooth muscle contraction' | 0.0803578 |
|  | 'Platelet activation' | 0.0523469 |
|  | 'Circadian entrainment' | 0.0175669 |
|  | 'Long-term potentiation' | 0.0336346 |
|  | 'Neurotrophin signaling pathway' | 0.0736238 |
|  | 'Glutamatergic synapse' | 0.0146391 |
|  | 'Cholinergic synapse' | 0.0298974 |
|  | 'Serotonergic synapse' | 0.0505563 |
|  | 'GABAergic synapse' | 0.0349762 |
|  | 'GnRH signaling pathway' | 0.0475486 |
|  | 'Estrogen signaling pathway' | 0.0809458 |
|  | 'Melanogenesis' | 0.0819526 |
|  | 'Thyroid hormone signaling pathway' | 0.0269076 |
|  | 'Oxytocin signaling pathway' | 0.0743192 |
|  | 'Glucagon signaling pathway' | 0.0271926 |
|  | 'Endocrine and other factor-regulated calcium reabsorption' | 0.0710498 |
|  | 'Gastric acid secretion' | 0.0244615 |
|  | 'Pancreatic secretion' | 0.0451711 |
| Huh-7 | 'Fatty acid degradation' | 0.0041178 |
|  | 'Valine, leucine and isoleucine degradation' | 0.0008841 |
|  | 'beta-Alanine metabolism' | 0.0169767 |
|  | 'Ether lipid metabolism' | 0.0838793 |
|  | 'alpha-Linolenic acid metabolism' | 0.0523739 |
|  | 'Propanoate metabolism' | 0.0012942 |
|  | 'Metabolic pathways' | 0.0005423 |
|  | 'Fatty acid metabolism' | 0.0856234 |
|  | 'PPAR signaling pathway' | 0.0806782 |
|  | 'Cytokine-cytokine receptor interaction' | 0.0509031 |
|  | 'Peroxisome' | 0.0134695 |

**Supplementary Table S7.** **Pathways enriched by genes upregulated in the resistance high-risk GC patients but downregulated in the HGC-27 cell lines treated by melatonin. p(<0.1) was adjusted by Benjamini and Hochberg.**

| Cell lines | Pathway Name | *p* |
| --- | --- | --- |
| HGC-27 | 'Purine metabolism ' | 1.89E-03 |
|  | 'Pyrimidine metabolism ' | 1.30E-03 |
|  | 'Ribosome biogenesis in eukaryotes ' | 8.84E-13 |
|  | 'RNA transport ' | 8.31E-09 |
|  | 'DNA replication ' | 2.40E-06 |
|  | 'Spliceosome ' | 3.98E-09 |
|  | 'Proteasome ' | 4.85E-04 |
|  | 'Base excision repair ' | 7.22E-05 |
|  | 'Mismatch repair ' | 3.19E-3 |
|  | 'Cell cycle ' | 3.63E-10 |
|  | 'Oocyte meiosis ' | 3.27 E-4 |
|  | 'Progesterone-mediated oocyte maturation ' | 2.44 E-3 |
